# Nonconvulsive status epilepticus manifesting as bradyphrenia: a case report

**DOI:** 10.4076/1757-1626-2-7069

**Published:** 2009-06-26

**Authors:** Martijn Weisfelt, Dick van den Wijngaard

**Affiliations:** Kennemer GasthuisBoerhaavelaan 22, 2035 RC, HaarlemThe Netherlands

## Abstract

Generalised convulsive status epilepticus continues to be a medical emergency with high morbidity and mortality. The patient with convulsive status epilepticus has continuous or rapidly repeating seizures. In contrast, symptoms in nonconvulsive status epilepticus are often more subtle which frequently delays the diagnosis. This case describes a 27 year-old man who presented after a first seizure and only displayed symptoms of slight bradyphrenia. An electroencephalogram revealed a generalised status epilepticus. As nonconvulsive status epilepticus may clinically display only subtle symptoms a high index of suspicion is needed to initiate electroencephalographic studies.

## Introduction

Generalised convulsive status epilepticus (GCSE) is considered a neurological emergency that requires prompt diagnosis and treatment, as delay is associated with a higher likelihood of poor response to treatment and worse outcome [1]. Whereas patients with GCSE have continuous or rapidly repeating seizures, nonconvulsive status epilepticus (NCSE) is characterized by behavioural or cognitive change for at least 30 minutes with electroencephalographic (EEG) evidence of seizures [2]. Because EEG is needed for diagnosis, only a high index of suspicion leads to a request for the study. Furthermore, the cognitive changes during NCSE are often erroneously ascribed to a postictal state, intoxication, psychogenic or psychotic states, and mental retardation [2].

## Case presentation

**Figure 1.**
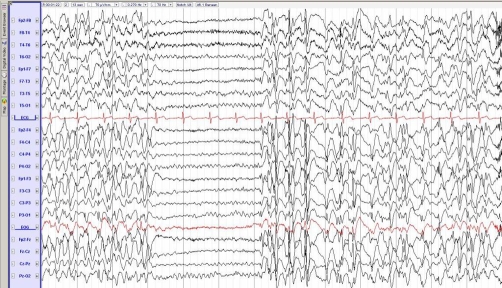
Electroencephalogram showing bilateral diffuse synchronous epileptic discharges (electrical status epilepticus).

A 27 year-old man presented with a first time seizure. He had telephoned his girlfriend earlier that evening claiming he was not feeling well. During this call the girlfriend suddenly heard a scream and a subsequent fall. When she arrived at the patient’s house 30 minutes later she found the patient lying on the ground. He was confused and had bitten his tongue. At arrival at the emergency room he was still somewhat disorientated. Past medical history revealed sleeping difficulties for which he used a benzodiazepine (oxazepam) and a possible seizure during his childhood. He used no other medication. His father was terminally ill of a brain tumour. Neurological examination showed slight bradyphrenia (slowness of thought processes) and agitation but revealed no further abnormalities. Computed tomography (CT) of the brain and results of laboratory studies showed no significant abnormalities. EEG the following day showed bilateral diffuse synchronous seizures (electric generalized NCSE; Figure 1). Despite the profound EEG abnormalities the patient was in relatively good clinical condition and he was considered to suffer from generalized NCSE. Therefore, the treatment with valproate 500 mg two times daily was initiated and the patient discharged himself later that day against medical advice to be with his dying father. Ten days later EEG was improved markedly and only revealed focal abnormalities on the left temporal region. His father had died three days earlier.

## Discussion

NCSE can occur in a variety of disorders, including hypoxia, metabolic disturbances, and after convulsive seizures [3]. Clinically patients may display subtle, intermittent focal or multifocal rhythmic movements suggestive of seizures; there may not be movements [3]. The wide overlap between the clinical symptoms of NCSE and many other disorders causing change in mental status limits the usefulness of clinical measures for diagnosis [4]. Therefore, the condition can be difficult to diagnose, especially in the obtunded/comatose patient in which comorbid condition are often present [5].

Treatment recommendations between GCSE and NCSE differ significantly. The preferred treatment pathway for GCSE is intravenous administration of lorazepam or diazepam directly followed by phenytoin or equivalent fosphenytoin. Patient with refractory GCSE should be treated in an intensive care units by anaesthetic doses of midazolam, propofol or barbiturates; the anaesthetics are titrated against a burst suppression pattern on EEG monitoring [6]. The initial therapy of NCSE depends on the type and the cause. In most cases of absence NCSE, a small intravenous (i.v.) dose of lorazepam or diazepam will terminate the attack. Complex partial NCSE is initially treated such as GCSE, however, when refractory further non-anaesthetising substances should be given instead of anaesthetics. In subtle SE i.v. anaesthesia may be required [7]. Regarding treatment, comatose NCSE patients treated with benzodiazepines may worsen, whereas generalized nonconvulsive status epilepticus patients may suffer iatrogenically from aggressive treatment (hypotension and respiratory depression) necessitating balancing the potential neurologic morbidity of NCSE against the possible morbidity of IV antiepileptic drugs. Therefore, less aggressive treatment with oral antiepileptics (e.g., Valproic acid) may be considered in clinically mild cases [2,7]. Determining the prognosis in nonconvulsive status epilepticus (NCSE) is complicated by several factors: under-recognition of NCSE with its spontaneous resolution; incorrect diagnosis of NCSE based on misinterpretation of EEG patterns as NCSE and grouping of different populations that have markedly different co-morbidities [5,9]. There are almost no prospective studies with premorbid neuropsychometric studies, and retrospective studies typically include isolated cases, or case series that include conditions in which the cause of NCSE itself causes cognitive morbidity. Larger, prospective studies will be needed to truly determine the prognosis in the different types of NCSE, stratified according to associated degrees of impairment [2].

## Conclusion

NCSE can occur in a variety of conditions and may clinically display only subtle symptoms. Because EEG is needed for diagnosis, a high index of suspicion is needed to initiate electroencephalographic studies.

## Abbreviations

CT: Computerized tomography
GCSE: Generalised convulsive status epilepticus
NCSE: Nonconvulsive status epilepticus
EEG: Electroencephalography
